# The ABCD approach for managing neuropsychiatric symptoms of dementia

**DOI:** 10.1097/01.NURSE.0000942784.14340.1f

**Published:** 2023-07-20

**Authors:** Amy Siple

**Affiliations:** **Amy Siple** is a national speaker on healthcare issues that impact older adults and the empowerment of healthcare givers. She has served the primary care needs of residents in long-term care as an NP for over 2 decades.

**Keywords:** Alzheimer disease, behavioral symptoms, dementia, neuropsychiatric symptoms, psychological symptoms, root cause analysis

## Abstract

The neuropsychiatric symptoms associated with dementia, often referred to as unwanted behaviors, are one of the most difficult aspects of this disorder for caregivers to navigate. This article presents strategies to manage dementia-related neuropsychiatric symptoms.

**Figure:**
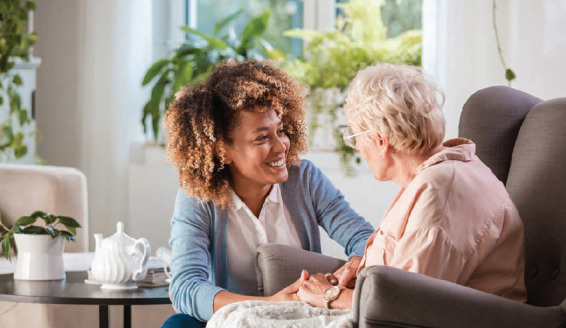
No caption available.

The predicted number of patients with dementia in the next 30 years represents a significant challenge to our healthcare system. It is estimated that 6.5 million had Alzheimer disease (AD), the most common type of dementia, in 2022 in the US; by 2060, this number is projected to be 13.8 million.^1^,^2^ Since AD represents only one type of dementia, the total number of people with dementia is likely underreported.

In 2021 more than 11 million people provided approximately 16 billion unpaid caregiver hours for those with dementia.^1^ Unfortunately, this represents a decline in those volunteering to render care.^2^ This volunteer decline is coupled with critical decreases in healthcare workers, particularly in the long-term care industry.^3^ Thus, nurses must focus on improving skills of efficacy and efficiency in caring for those with dementia.

One of the biggest challenges when caring for someone with dementia is managing the accompanying neuropsychiatric symptoms, often called “behaviors.” These include yelling, pacing, delusions, and aggression.^4^ There are two primary approaches to management. The first is medication-based; the second involves searching for the root cause or etiology of the behavior and intentionally equipping caregivers with management strategies. This article explores these strategies through a representative series of case examples. Specific patient identifiers have been altered. Any similarity to actual persons, living or deceased, is purely coincidental.

## The Pharmacologic Management Approach

Psychotropic medications have often been used off-label to manage neuropsychiatric symptoms associated with dementia.^5^ This practice occurs despite evidence showing limited benefits and high mortality risk.^6^ Patients with dementia are often sedated with several pharmacologic agents to reduce agitation and other neuropsychiatric symptoms.

### Case Example 1

*An NP practiced the medication-based approach to managing the neuropsychiatric symptoms associated with dementia. A particularly challenging patient would bite, kick, scratch, and spit on staff during personal care. She was generally sedated with a benzodiazepine before bathing. On one occasion, medicating her resulted in a fall, causing a forehead laceration that required sutures. When paged about this incident, the NP was rounding with a physician. He accompanied the NP and modeled engaging in a root cause analysis approach to understand the patient's unwanted behaviors. The NP and physician discovered that the patient was raped in her youth, and the direct care staff providing bathing services were primarily males of the same ethnic background as her attacker. When the physician approached the patient, he sat down to make eye contact with her and lowered his voice and tone. He dimmed the lights and played soft music. The NP watched the patient de-escalate dramatically; the NP injected local anesthetic and repaired the laceration without difficulty*.

This case highlights the need to engage in trauma-informed care. Up to 90% of older adults have experienced at least one traumatic event.^7^ Some will develop triggers, a stimulus that activates a painful memory or emotion, that can result in secondary traumas. Patients with dementia seem to be especially vulnerable.^8^ They can find themselves in a loop in which the traumatic event replays in their mind. The use of psychotropic agents in this case example resulted in a fall with injury.^9^ This event created additional trauma for the patient and increased the needed caregiver hours.

Numerous psychological symptoms result from chronic cognitive impairment, including anxiety, depression, fear, and anger.^10^ A caregiver's ability to imagine themselves in the patient's position can help them gain understanding and formulate an empathetic response.

Expressive and receptive aphasia are common features of dementia.^11^ For example, communicating fecal impaction without words could become impossible, even for those with a fully functioning brain. Unwanted behaviors are typically an expression of unmet needs.^12^

The nurse should strive to be the best detective possible when searching for a root cause when patients with dementia exhibit unwanted behaviors. This will require a multifactorial approach.^10^

The analysis starts with the first phase of the nursing process: assessment. Although the formulation of a nursing diagnosis depends on objective and subjective data, 80% of the information required for a diagnosis generally comes from the patient history.^13^ Obtaining historical data from patients with dementia is often challenging because of cognitive symptoms such as memory impairment, confusion, and the patient's inability to relay the symptoms they experience. Engaging the caregivers in this process is, therefore, essential.

### Case Example 2

*As a former professional football player, Joe was an imposing force. His massive frame dwarfed everyone else in his memory care unit. He often became physically aggressive when frustrated. Psychotropic agents were a cornerstone of his plan of care*.

*One day, a physician entered Joe's room. Joe was sleeping, and the physician attempted to wake him by turning on the room light and rocking him back and forth. Joe opened his eyes and turned to look at his physician. Joe appeared confused and did not answer any of the questions being posed to him, like “How are you?” and “Do you have any pain?” He remained still as his physician auscultated his heart and abdomen while maintaining a perplexed expression. When his physician turned towards the door, Joe got out of bed. The physician turned around, and Joe grabbed him by both shoulders and pinned him against the wall. Joe did not say anything during this encounter*.

*The physician remained calm and spoke in a relaxed tone. A nurse in an adjacent room entered and told Joe, “It is okay. You are safe. I need you to let go of him.” Joe released the physician and took the nurse's hand as she led him back to his bed*.

*A few weeks after the incident with his physician, Joe was transferred to an NP's care. His body odor was readily apparent. Staff confessed Joe had not bathed in two and a half months. They said they offered bathing services, but he declined*.

*After reviewing his history and care plan, the NP approached Joe and began an assessment of his ankles and knees*.

*The NP asked about football injuries to these joints, which he confirmed. Then the NP asked, “Did coach ever make you get in the whirlpool for your ankles and knees?” He affirmed. The NP responded, “Joe, coach says we need to get in the whirlpool.”*

*“Coach says?”*

*“Coach says.”*

*“Well, okay. If coach says.”*

*The NP asked a couple of caregivers to accompany her to the bathroom. After removing Joe's shoes and socks, she helped him move into the tub. He said, “I am going to get my pants wet.”*

**Table:** The ABCD approach^11^

A	Antecedents	What happened prior to the unwanted behavior?
B	Behavior	Describe in detail the exact events as they happened.
C	Consequences	What was done in response to the behavior and did it escalate or de-escalate the patient?
D	Decision/Debrief	What did caregivers learn from this encounter that can shape future responses?

*“You are right, Joe. Let's take those off and your skivvies too.” The NP said nothing about his shirt. He complied and got in the tub. The NP started the water, eventually reaching his shirt's level. She calmly asked, “Do you want me to help you get your shirt off so it doesn't get wet?” He complied, and the NP was able to complete the bath*.

*Upon assuming Joe's care, the NP noted he was on an atypical antipsychotic agent and a routine and p.r.n. benzodiazepine. One of the adverse reactions of the antipsychotic agent was pseudoparkinsonism (drug-induced Parkinson Disease). In response, carbidopa/levodopa was added to his medication regimen. The antipsychotic agent had also caused drug-induced diabetes, for which metformin was added. Joe developed diarrhea after starting the metformin and insomnia and headaches after starting the carbidopa/levodopa. His diarrhea was treated with loperamide; the insomnia was treated with trazadone; and ibuprofen was given for the headaches. Although the ibuprofen was only listed as p.r.n., the staff gave it at least twice daily to treat headaches and pain secondary to osteoarthritis. Joe developed gastrointestinal bleeding; thus, a proton pump inhibitor (PPI) was needed. In addition, Joe had pneumonia twice in the past year. Benzodiazepines and PPIs are both linked to the risk of pneumonia*.^14^,^15^ *In reviewing Joe's history, the NP recognized that polypharmacy was a strong contributor to his comorbidities and likely exacerbated his dementia-related neuropsychiatric symptoms*.

In the ABCD Approach, the letter A stands for antecedents or activating events.^16^ This involves exploring what happened before the reported behavior to search for a cause and effect.

The letter B represents the behavior that is described in detail. Nurses should report the behaviors observed, such as Joe's agitation, and precisely what the nurse saw and heard.

The letter C stands for consequences. What did the healthcare personnel (HCP) do, and how did it impact the patient's behavior? Did the HCP's response reinforce or deter the situation? Did the HCP escalate or de-escalate the tension with their actions?

Lastly, the letter D stands for deciding and debriefing. Everyone involved should be part of the debriefing and be encouraged to present their perspective on what happened. Nurses should consider how to communicate this information to other caregivers. There was an initial plan to send Joe to a behavioral therapy unit at the hospital. However, following the debriefing, staff agreed they would avoid waking Joe unless necessary. It seemed especially important to prevent startling Joe, which was incorporated into the care plan. Joe responded well to a calm and relaxed demeanor, the assurance of safety, and saying, “I need you to let go.” These are all behaviors caregivers can easily replicate and should be made a part of the care plan. Unfortunately, in this scenario, Joe was given a benzodiazepine agent following the altercation, which produced sedation, causing him to sleep for the rest of the morning. The caregiver's nonpharmacologic response was very effective in de-escalating Joe. Staff reported the decision to offer the p.r.n. benzodiazepine was based on their fear after witnessing the violent interaction. It would be worthwhile to avoid benzodiazepine and focus on nonpharmacologic interventions (see *The ABCD approach*).

Information from the patient's history can be used to formulate creative responses to neuropsychiatric symptoms and de-escalate tense situations. In this case, knowledge of Joe's respect for his coach was used to encourage his compliance with personal care. However, it may not always be effective, and caregivers must be flexible.

Eliminating the neuropsychiatric symptoms associated with dementia is unrealistic because patients with moderate to late-stage dementia cannot learn new coping skills.^10^,^12^ However, equipping caregivers with information and tools that promote meaningful interactions may reduce caregiver burden and boost caregiver confidence.^17^,^18^ Many caregivers of patients with dementia find themselves frustrated and discouraged. They report feeling ill-prepared to understand and deal with the neuropsychiatric symptoms associated with dementia.^17^,^18^ As a result, they may depend on psychotropic agents to sedate those with dementia.^19^ The Centers for Medicare and Medicaid Services strongly discourages the off-label use of antipsychotic agents to manage behaviors in patients with dementia and emphasizes gradual dose reductions and cessation when possible.^20^ Mortality may be 60% higher in patients with AD taking antipsychotic agents; that risk could double if they are on two or more antipsychotics.^21^ This increase in mortality has been shown to persist over at least 12 months.^22^ In 2005, the FDA issued a boxed warning for antipsychotics in patients with dementia due to fall risk, movement disorders, and death.^22^ The 2019 BEERS criteria stated that antipsychotics should be avoided to treat neuropsychiatric symptoms associated with dementia unless nonpharmacologic interventions have failed and the patient is a threat to themselves or others.^23^ While the overuse of antipsychotic agents has increased morbidity and mortality, they do have their place in managing some patients with dementia, particularly those with mania or psychosis.^24^ In fact, brexpiprazole, an antipsychotic, was recently approved for Alzheimer's dementia-related agitation through a fast-track process by the FDA. This drug has a boxed warning for increased mortality, as do all antipsychotics used in patients with dementia.^22^,^25^ Weighing risks against possible benefits requires extreme caution.

Benzodiazepines are used routinely and intermittently in patients exhibiting neuropsychiatric symptoms associated with dementia.^19^,^26^,^27^ They have been shown to increase the risk of dementia, and further exacerbate neuropsychiatric symptoms associated with dementia. The BEERS criteria included a strong recommendation in 2019 to avoid these agents for treating insomnia, agitation, or delirium.^23^ Despite this recommendation, agitation is one of the top reasons cited in long-term care centers for using these agents.^27^ Rebound insomnia and rebound anxiety are well-established risks associated with benzodiazepines.^28^ Benzodiazepines can also cause delirium and carry a risk of dependence. The most significant concern, however, is the high fall risk associated with benzodiazepines when used in older adults.^29^

The use of psychotropic agents in treating the neuropsychiatric symptoms associated with dementia contributes to polypharmacy. Additional agents may be added to treat the adverse events associated with the psychotropic agents, further compounding the problem. The risk of drug-to-drug interactions is substantial in those taking numerous agents. Studies show the risk could be as high as 80% for those on seven or more medications.^30^

The pathophysiologic process of dementia affects multiple body systems. For example, aphasia, visuospatial deficits, and apraxia are common features for individuals with Alzheimer's dementia.^31^ Therefore, an interdisciplinary approach to neuropsychiatric symptoms associated with dementia is an essential component of the care plan.^32^

## Conclusion

Neuropsychiatric symptoms should be expected in patients with dementia. The most effective mitigation strategy is searching for the etiology or unmet need that prompts unwanted behaviors. Key nursing considerations include understanding the patient's history, provoking factors, and using the ABCD approach.

Equipping caregivers with the tools, resources, and skills necessary to mitigate neuropsychiatric symptoms must become a priority. Psychotropic agents may have a limited role in managing behaviors, and a careful risk versus benefit analysis needs to occur before they are prescribed. An interdisciplinary approach is key in managing dementia-related neuropsychiatric symptoms.

## The ABCD approach in action

An NP was recruited to assist a long-term-care facility trying to reduce psychotropic agent use in their patients. When benchmarked against other facilities, the facility had a 61% ranking for the use of antipsychotic agents and 90% for the use of antianxiety and hypnotic agents. CMS had notified them that their high psychotropic usage could negatively impact reimbursement. The NP assumed the primary care of their residents and equipped the other nurses and patient-care staff with the information and tools necessary to mitigate the neuropsychiatric symptoms associated with dementia. During the NP's weekly visits, she asked staff to notify her if there were unwanted behaviors so she could observe them. Not only did she want to understand what was happening (ABCD approach), but she also sought opportunities to model de-escalation techniques. In addition, she offered free classes to staff on the risks associated with psychotropic agents and tips for managing dementia-related behaviors. Phone consultations with the pharmacist, activity directors, social workers, therapy staff, and families helped ensure an interdisciplinary approach. The results were dramatic: In 5 months, antipsychotic usage dropped from 61% to 9%, and antianxiety and hypnotic usage declined from 90% to 19%. Interestingly, the report of unwanted behaviors dropped from 85% to 59%.^33^ It is difficult to determine if the number of unwanted behaviors decreased or if the staff's perception of them decreased.
